# Modeling the Afferent Dynamics of the Baroreflex Control System

**DOI:** 10.1371/journal.pcbi.1003384

**Published:** 2013-12-12

**Authors:** Adam Mahdi, Jacob Sturdy, Johnny T. Ottesen, Mette S. Olufsen

**Affiliations:** 1 Department of Mathematics, North Carolina State University, Raleigh, North Carolina, United States of America; 2 Department of Science, Systems, and Models, Roskilde University, Roskilde, Denmark; Indiana University-Purdue University Indianapolis, United States of America

## Abstract

In this study we develop a modeling framework for predicting baroreceptor firing rate as a function of blood pressure. We test models within this framework both quantitatively and qualitatively using data from rats. The models describe three components: arterial wall deformation, stimulation of mechanoreceptors located in the BR nerve-endings, and modulation of the action potential frequency. The three sub-systems are modeled individually following well-established biological principles. The first submodel, predicting arterial wall deformation, uses blood pressure as an input and outputs circumferential strain. The mechanoreceptor stimulation model, uses circumferential strain as an input, predicting receptor deformation as an output. Finally, the neural model takes receptor deformation as an input predicting the BR firing rate as an output. Our results show that nonlinear dependence of firing rate on pressure can be accounted for by taking into account the nonlinear elastic properties of the artery wall. This was observed when testing the models using multiple experiments with a single set of parameters. We find that to model the response to a square pressure stimulus, giving rise to post-excitatory depression, it is necessary to include an integrate-and-fire model, which allows the firing rate to cease when the stimulus falls below a given threshold. We show that our modeling framework in combination with sensitivity analysis and parameter estimation can be used to test and compare models. Finally, we demonstrate that our preferred model can exhibit all known dynamics and that it is advantageous to combine qualitative and quantitative analysis methods.

## Author Summary

Many people have experienced lightheadedness when standing up, yet the exact cause of this phenomenon remains unknown. For some people, lightheadedness occurs because of anomalies in the blood pressure control system, which keeps blood flow and pressure at homeostasis. One way to explore this system is via mathematical modeling, which can offer valuable insights into the complex dynamic processes. This study develops a framework for modeling activity of the baroreceptor neurons. The models consist of three components reflecting three physiological mechanisms relating blood pressure to the baroreceptor firing rate: modulation of arterial blood pressure causes dilation of the arterial wall, stimulating mechanoreceptors within the baroreceptor nerve endings, emanating from the aortic arch and carotid sinus, which in turn modulates the firing rate of the baroreceptor neurons. This signal is integrated in the brain stem, stimulating baroreflex efferents to counteract the pressure increase. In this study, we review the main static and dynamic features of the baroreceptor firing activity, and show, using a combination of modeling techniques and rat aortic baroreceptor data, how to build a computationally efficient, yet biologically correct model. These models are important components for describing efferent responses, such as: heart rate, contractility or stroke volume.

## Introduction

**Figure 1:**
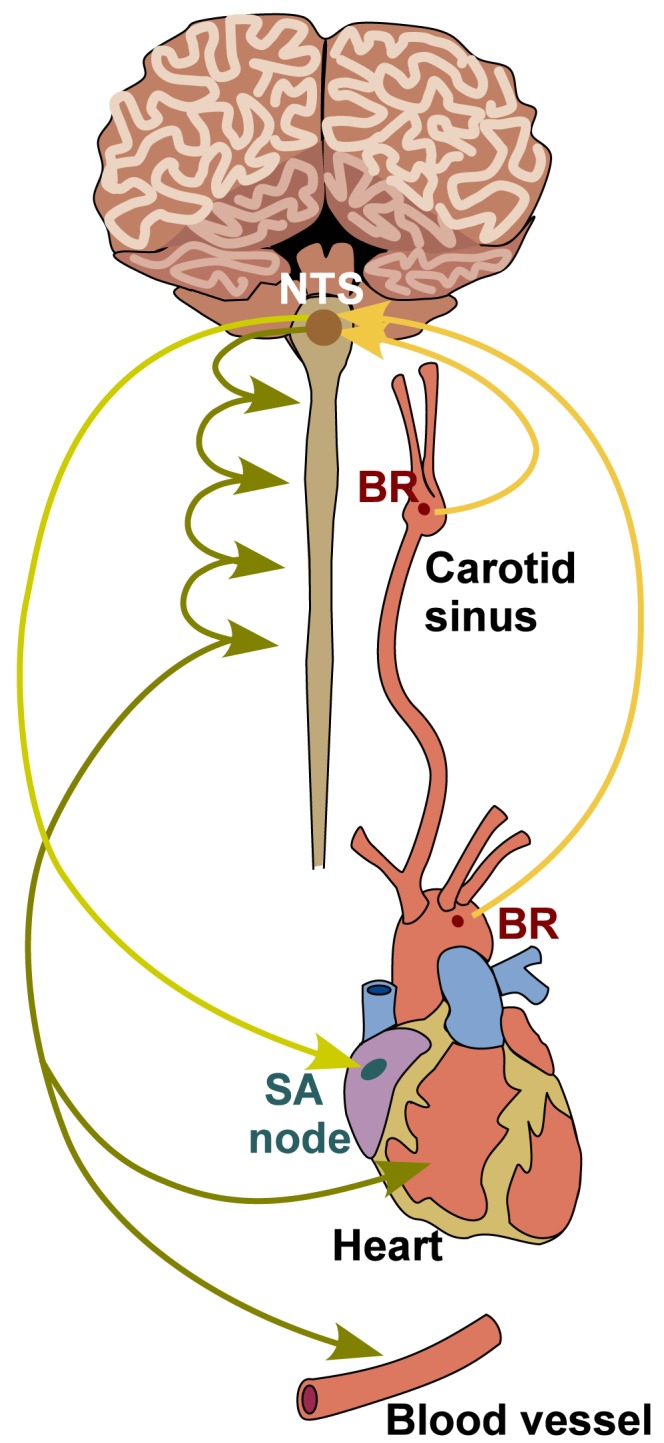
Schematic representation of the BR feedback system. Stretch sensitive BR neurons originate in the carotid sinuses and the aortic arch. In these arteries, dynamic changes in blood pressure cause vessel deformation, modulating stretch of mechanoreceptors channels found in the BR nerve endings. Stimulation of these receptors modulates frequency of action potential formation, a signal integrated in the NTS. From the NTS, efferent sympathetic and parasympathetic outputs are generated determining the concentrations of neurotransmitters acetylcholine and noradrenaline, which stimulate or inhibit heart rate, cardiac contractility, vascular resistance and compliance, the latter via activation of smooth muscle cells constricting or dilating the radius of arteriolar vessels.

The main role of the cardiovascular (CV) system is to provide adequate oxygenation of all tissues, a function which is achieved by maintaining homeostasis of blood flow and pressure. When a mammal is subjected to an orthostatic maneuver (e.g., running, jumping, etc.), its blood volume is redistributed, moving the system state away from homeostasis [1]. To re-establish homeostasis a number of control mechanisms are activated regulating vascular resistance and compliance, and cardiac pumping efficiency and frequency. An important contributor to this control system is the *baroreflex*, which uses specialized neurons called baroreceptors (BR) for signaling [2]. The BR neurons originate in the arterial wall and terminate in the nucleus solitary tract (NTS), where sensory information is integrated. These neurons are continuously stimulated via activation/inhibition of mechanosensitive receptors responding to changes in arterial wall stretch imposed by pulsating blood pressure [3]. This stimulus modulates the formation of action potentials propagating along the BR nerves terminating in the NTS, where efferent signals are generated to regulate heart rate, cardiac contractility, as well as vascular resistance and compliance. It is known that the baroreflex system contributes to short-term blood pressure regulation, operating on a time-scale of seconds to minutes [4]. For example, upon head-up tilt, blood is pooled in the lower extremities, increasing blood pressure in the lower body, while decreasing it in the upper body, causing an imbalance, which persists until the baroreflex system is activated. Figure 1 shows a schematic representation of the baroreflex pathways. While the BR pathways are generally well established, analysis of the complete control system, including afferent and efferent signaling, is hindered by the difficulty of measuring the activity of each component without disrupting the feedback loop. For example, *in vivo*, only macroscopic quantities can be measured non-invasively including heart rate and blood pressure. From such measurements it is difficult to examine how the individual components of the system interact and consequently it is difficult to determine which sub-systems are compromised in subjects experiencing baroreflex failure [5] or decreased arterial baroreflex sensitivity [6]. These difficulties limit the development of targeted diagnosis procedures and treatment plans aiming to alleviate symptoms for patients.

Mathematical modeling is an eminent tool for gaining more insight into this complex feedback loop, offering a stringent and systematic way to identify underlying mechanisms of the system. For example, the only way to estimate model parameters and thereby suggest essential biomarkers, which may not be directly measurable, is by using models in combination with direct measurements. Modeling also offers a way to understand complex systems, as it makes the inaccessible accessible, a concept denoted the “mathematical microscope” [7].

This paper focuses solely on the afferent part of the baroreflex system, while future studies will address efferent signaling and integration of the two parts within a system level model. Since the 1950s researchers have put forward numerous mathematical models [8]–[19], which tried to integrate known dynamics with hypothesized mechanisms in order to provide more understanding of the system as a whole. Many insights have been gained, however, most of these models were developed to describe BR response to a particular stimulus, rather than to a range of stimuli eliciting all known responses. Therefore they all lead to different hypotheses explaining the system mechanisms. Inspired by shortcomings of previous studies, we developed a *modeling framework* containing model components reflecting physiological pathways. This framework splits the afferent signaling into three parts describing vessel wall deformation, mechanoreceptor stimulation, and the frequency of action potential generation. For each component we propose multiple models, which we test both qualitatively and quantitatively. This new approach allows us to understand the contribution of each component model to the overall signal. For example, if the objective is to build a BR model that can reflect the response to a sinusoidal pressure stimulus observed experimentally, the modeling framework can be used to identify which combinations of components are sufficient to describe the experimental outcome, and which component models may be excluded from possible explanations of observed features. Moreover, we show how our framework may be used to inform hypotheses, by suggesting a particular component mechanism responsible for generating a given pressure-response feature of BR firing.

## Methods

### Experimental data and its features

In this section we describe the main *qualitative* characteristics of BR firing rate as well as the data used for *quantitative* model tests.

#### Qualitative features of the BR firing rate

**Figure 2:**
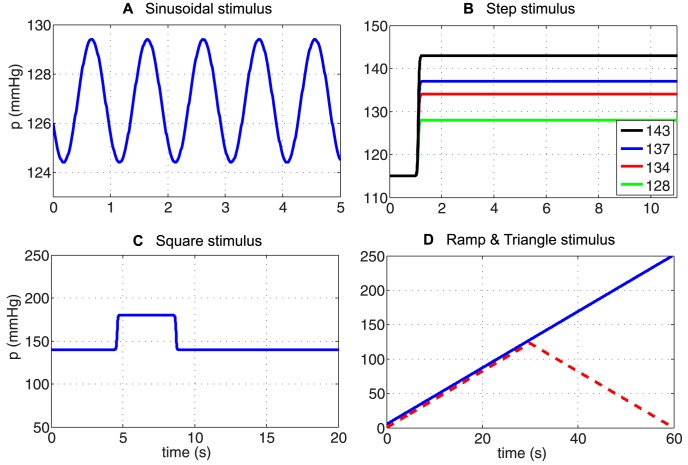
Various types of BR input pressure. To test our models we applied a number of pressure stimuli: (A) sinusoidal, (B) step increases, (C) square (step increase followed by a step decrease), (D) ramp and triangular. The above stimuli were used for testing the models' responses both qualitatively and quantitatively.

Although BR firing patterns depend on the type of BR, e.g., whether they are connected to myelinated or unmyelinated axons [20], there are a number of features nearly all BR neurons exhibit. We characterize these according to observations obtained by stimulating isolated rat aortic BR neurons with a range of pressure stimuli including: sinusoidal, step increases and decreases, and ramp increases and decreases (Figure 2). The most commonly noted features of the BR response to imposed pressure stimuli include: saturation and threshold, adaptation and overshoot, as well as post-excitatory depression and rectification. Below, we describe each of these firing rate patterns in more detail.

*Threshold*. Observed in response to a step or ramp increase in pressure. This phenomenon was first described by Bronk and Stella [21], [22] in the 1930s. They observed that a small step increase from a given baseline blood pressure did not trigger BR firing, but when the pressure was increased above a certain threshold, the BR nerve began to fire continuously. The threshold was later observed to increase with an increased baseline pressure [23]–[26]. Moreover, Seagard et al. [27] observed that the type of baroreceptor (myelinated or unmyelinated) strongly affects the threshold pressure. The precise mechanisms underlying the threshold phenomena remains unknown, but it is thought to be attributed to the characteristics of ion channels associated with generation of action potentials [28].

*Saturation*. Observed in response to a ramped increase of blood pressure. As the pressure is increased linearly, the BR firing rate first increases almost linearly (with pressure). Then, at a given frequency, the firing rate approaches some limiting value (the saturation level) [23]. This phenomenon was also observed by Bronk and Stella [21], [22]. They noted that for normotensive rabbits, the firing rate saturates around 120–140 Hz. Later, Seagard et al. [27] studied saturation by stimulating a single carotid BR nerve fiber, extracted from a mongrel dog, with a slow linearly increasing pressure. This experiment showed firing rate saturation at 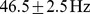. These observations led to the separation of nerves as type I (large myelinated aortic (A) nerve fibers) and type II (smaller aortic (A) and unmyelinated carotid (C) nerve fibers). They observed type I BR neurons displayed a discontinuous firing pattern, characterized by a sudden onset of discharge at the average threshold pressure of 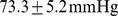, whereas type II neurons displayed a continuous, sigmoidal firing pattern saturating at 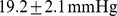.

*Overshoot and adaptation*. Observed in response to a step change in pressure. The firing rate responds by immediately increasing the rate of discharge, followed by a slow adaptation to a new lower steady state value. Brown et al. [29, Figure 5] noted that the relationship between the size of the overshoot and the level of the pressure stimulus is almost linear. The adaptation level depends on the magnitude of the pressure change. This phenomenon was first observed by Landgren [8, p. 7], who discovered that 50% of adaptation occurs within 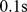 following the the pressure stimulus, 95% is completed after 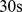, whereas full adaptation requires a very long time, more than 2 min. It was later confirmed by Srinivasen and Nudelman [11] and Brown et al. [30], though from these later studies it is not clear that adaptation requires three distinct timescales. Moreover, Brown [30] noted that the frequency of the adapted firing rate is the same whether the baseline pressure level is reached from a higher or a lower pressure level. Several studies have observed that the level of the steady-discharge is proportional to the applied pressure [20], [29]. No mechanism has been established as the cause of adaptation; however, Franz et al. [31, p. 823] propose viscoelastic relaxation as the source of adaptation in the firing rate.

*Post-excitatory depression* (PED). Observed following a step-decrease in pressure. In response to this stimulus the BR firing ceases for a short period, after which it recovers to a rate corresponding to the newly established pressure level. While the term PED was put forward by Brown et al. [30], [32], who studied the phenomena extensively, it was first observed by Bronk and Stella [21] when they noticed that BR firing ceased during diastole. Later, Wan et al. [33] observed that the length of the pause depends on the depth of the pressure drop. Brown [32, p. 504], suggested that an electrogenic-sodium pump could be the potential mechanism for this phenomena.

*Asymmetry (or hysteresis)*. Observed following a sequential rise and fall of blood pressure (see sinusoidal, square, and triangular stimulus shown in Figure 2). This phenomenon was described by Katona and Barnett [34], but have also been discussed by Coleridge, Angell, Pelletier et al. [23], [35]–[37]. These studies all observed that the BR firing rate exhibits asymmetrical responses to rising and falling blood pressure. However, asymmetry can be observed in response to any stimuli involving a symmetric increase and decrease in pressure. Thus it may also be observed in PED and in response to periodic sinusoidal forcing. In the time-domain, it may not be easy to see that a sinusoidal stimulation leads to asymmetry, but it can be observed by depicting BR firing as a function of pressure, which gives rise to hysteresis loops. This phenomenon is closely related to adaptation and overshoot, thus viscoelastic relaxation exhibited by the arterial wall, could explain its origin.

#### Description of experimental data

So far we have focused on describing the qualitative features of the BR firing rate. However, if the objective is to understand how these responses are modulated in disease or between species it may be important to predict the BR firing rate quantitatively.

Below we describe the main features of data used for quantitative predictions. Data were obtained by digitizing results reported by Brown et al. [20] and Saum et al. [32]. From these studies we extracted data from a total of six experiments, grouped with respect to the applied pressure stimulus: sinusoidal, step increase with four different amplitudes, and a square pulse. These stimuli are depicted in Figure 2A–C.

*Sinusoidal pressure stimulus*. To test the models' abilities to mimic *in vivo* dynamics, we used data reported by Brown et al. [20, Figure 2A]. They stimulated the stretch-sensitive receptors using a sinusoidal pressure stimulus mimicking the natural blood pressure rhythm and recorded the corresponding BR firing rate. Several studies [9], [17], [38]–[40] have reported similar experiments. This type of data allows us to evaluate whether the model can exhibit asymmetry and rectification. The study [20] reports firing rate responses recorded from 11 experiments using myelinated aortic BR axons extracted from Wistar-Lewis strain normotensive rats aged 4–6 months. For each experiment the neuron was stimulated using sinusoidal pressure wave with a frequency of 20 Hz, an amplitude of 5 mmHg, and a mean pressure of 127 mmHg. Steadily oscillating pressures were recorded over a period of 5 seconds. More details about experimental preparation can be found in [29], [32]. To obtain a smooth input stimulus, we fit the data to a sinusoidal function of the form
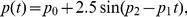
(1)where 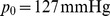. We estimated parameters 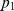 and 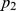 using the initial values 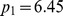 and 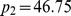 both radians/s.

*Multistep pressure stimulus*. To demonstrate overshoot followed by adaptation, we digitized BR firing rate data reported in [20, Figure 5]. This study shows BR discharge in response to four pressure step increases from a baseline pressure of 115 mmHg. The four step-increase stimuli are: 13 (to 128), 19 (to 134), 22 (to 137), and 28 (to 143) mmHg (Figure 2B). Experiments were done over a period of 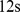, allowing the BR firing rate to adapt to a new steady level of discharge. In this study we used data reported by Brown et al. [20], though several experimental studies have reported similar observations [29], [41]. It should be noted, that no graph depicts the pressure stimulus. Brown et al. [20] reported the baseline pressure as well as the level of the pressure increase, but not the exact time denoting the onset of the stimulus. We modeled the stimulus using a smooth function of the form
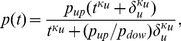
(2)where 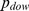, 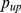 (mmHg) denote the baseline pressure and the increased pressure, respectively; 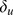 (s) denotes the onset of the pressure step increase, and 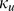 denotes the steepness of the increase. For the dataset under consideration the values 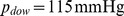, and 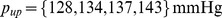 were taken from [20], while we estimated 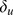 and 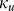. Initial values for these parameters were set to 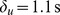 and 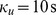 approximating the onset described in the experiment [20, Figure 5].

*Square pressure pulse stimulus*. To capture PED, we digitized data reported in Saum et al. [32, Figure 1], which examined PED and adaptation in slowly adapting aortic BR neurons extracted from normotensive and spontaneously hypertensive rats. Though this phenomenon has also been reported in several other studies including [8], [31]–[33], [42]. The study by Saum et al. [32] stated that PED could be elicited either mechanically by employing single or double pressure steps, or electrically by stimulating myelinated aortic BR axons extracted from normotensive Wistar-Lewis rats aged 4–6 month. This data shows a steady state discharge was elicited by stimulating the nerve with a baseline pressure of 140 mmHg. After 4 s the pressure was increased by 40 mmHg to 180 mmHg for a period of 4 s, after which it was reset to the baseline pressure of 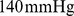. To allow the neuron to fully recover following the pressure drop, data were recorded over a period of 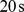. In order to avoid the problem of non-differentiability we modeled the pressure stimulus using the smooth function
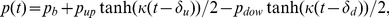
(3)where 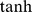 is the hyperbolic tangent. For this stimulus we used 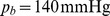, 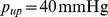, 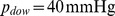, 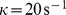, while 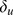 (s), 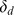 (s) were estimated.

### Models

**Figure 3:**
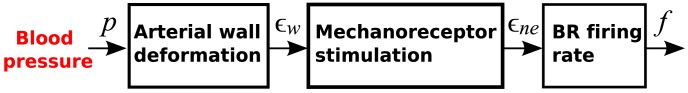
Block diagram used to describe the BR firing in response to an applied blood pressure stimulus. Applied changes in blood pressure induce changes in the arterial wall strain, which induce changes sensed by stretch sensitive mechanoreceptors found in BR within the arterial wall. This stimulus modulates frequency of action potential formation, which can be used to determine the BR firing rate.

To model the dynamics, which produce the BR firing rate in response to given blood pressure stimuli, we include three components separating distinct physiological pathways, and for each component we develop a number of linear and nonlinear models. The three components (Figure 3) include: arterial wall deformation, mechanoreceptor stimulation, and action potential generation. As a driving force for the models we use arterial pressure, which determines arterial wall deformation quantified by the wall strain. The wall deformation stimulates the stretch sensitive mechanoreceptors found in the BR nerve endings within the arterial wall. Thus changes in blood pressure modulate the opening of these channels, and thereby the current flowing through them, which determine the rate at which action potentials are formed. The time between subsequent action potentials determines the firing rate, and thus our models relate the receptor strain to the frequency of action potentials, thereby allowing us to predict the BR firing rate. For each model component, described below, we review previous modeling methodologies and use these to inform the design of the new component models, collectively used to describe the firing rate of afferent BR neurons in response to an applied blood pressure stimulus.

#### Arterial wall deformation

BR nerves originate in the wall of the the aortic arch and the carotid sinus and terminate in the NTS [43]. Action potentials transmitted along these nerves are generated by stimulation of mechanoreceptors found in the wall. These nerves are stimulated by pressure pulses passing through the vessel, and their firing patterns are modulated in response to changes of the frequency and magnitude of the pressure stimulus. It is well known [44] that the arterial wall deforms viscoelastically, though little is known about how this deformation impacts stimulation of the mechanoreceptors. This section describes models predicting the vessel strain as a function of blood pressure, while the next section describes characterization of mechanoreceptor stretch, which in turn modulates BR firing rate.

A detailed description of the arterial wall strain requires complex, anisotropic, viscoelastic models, accounting for dynamics associated with each layer of the wall as well as the interaction between the layers [44]. While such models can provide detailed description of wall deformation, without additional data they are not suitable for integration in higher-level models determining the BR firing rate. Another class of models are those assuming that the arterial wall is isotropic. These models represent the wall as a thin shell, and since arteries are tethered in the longitudinal direction, viscoelastic deformation is dominantly in the circumferential direction (cf. [45]). Such models determine the cross-sectional strain of the arterial wall in response to induced changes in applied stress, corresponding to the blood pressure [46]. Again, depending on the fidelity needed, these “stress-strain” models can be simplified. The simplest stress-strain models ignore viscous deformation and treat the wall as purely elastic. The stress-strain relationship may be either linear or nonlinear. In this study we consider three wall models, of which one is linear and elastic (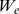, subscript 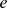 for elastic), one is linear and viscoelastic (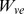, subscript 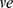 for viscoelastic), and one is nonlinear and elastic (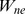, subscript 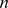 for nonlinear and 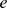 for elastic).

*Linear elastic wall model (* 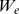 *)*. For a thin walled elastic vessel with an isotropic wall, neglecting the axial deformation, the wall strain 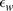 can be computed using *Laplace's law*,
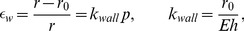
(4)where 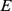 (mmHg) denotes Young's modulus, 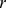 (mm) the vessel radius, 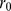 (mm) the unstressed radius at zero transmural pressure, and 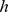 (mm) the wall thickness.

*Nonlinear elastic wall model (* 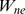 *)*. It is well known that the area-pressure response curve is nonlinear and can be modeled using a sigmoidal function, accounting for saturation of the vessel wall deformation at both high and low pressures. Following [46], [47] the pressure-area relationship can be modeled as
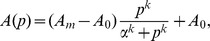
where 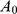 and 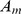 (mm^2^) are the unstressed and maximum cross-sectional area; 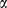 (mmHg) is the characteristic pressure at which the vessel starts to saturate; and 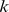 determines the steepness of rise of the sigmoidal curve, representing the stiffness in the lumen distention due to changes in pressure. Using (4) as a definition of wall strain 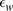, we obtain
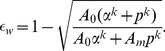
(5)


*Viscoelastic wall models (* 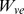 *)*. While the main contribution to arterial wall deformation is elastic, as mentioned above, the arterial wall is composed of tissue that has viscoelastic properties. Viscoelastic models encompass both elastic deformation and viscoelastic creep, and thus can be described using either linear or nonlinear elastic responses.

Linear viscoelastic response of the arterial wall is typically, although not solely, described using a number of springs (elastic elements) and dashpots (viscous elements) in various configurations. The so-called standard linear solid (SLS), is one of the most commonly used examples of such configurations. It involves a Maxwell element (a spring 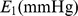 and dashpot 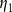 (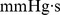) in series) in parallel with a spring 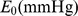. It is easy to establish that the total stress-strain relationship is given by
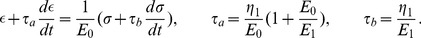
(6)To apply the SLS model to the arterial wall, we think of 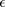 as vessel distention 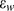 and the stress as the blood pressure 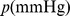. Moreover, assuming the arterial wall is a thin-walled elastic tube we can substitute 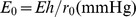 and obtain
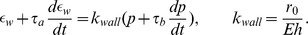
In order to avoid numerical differentiation of the data, following [46] we apply the integrating factor and transform this equation to
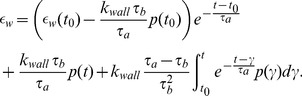
(7)


*QLV framework*. Formulated as linear elements in series and parallel, the above model cannot directly be extended to account for nonlinear elastic response; moreover, it is limited to models described using a finite number of components. It was noted by Fung [45], that biological tissues are not elastic and that strain history affects the stress. These tissues also exhibit a difference in the stress response between loading and unloading. Generalizing linear viscoelastic theory, Fung [45], introduced the so-called quasi-linear viscoelastic theory (QLV), which has been used successfully to model stress-strain relationships involving living tissues [48], [49]. The QLV theory is a flexible framework that includes linear viscoelastic theory and provides a more accurate description of the pressure-strain curve, especially in living tissues. We proceed with the assumption that the arterial wall can be modeled as homogeneous and isotropic thin walled cylindrical vessel [50]. Therefore the wall strain as a function of pressure can be determined as
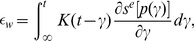
(8)where 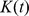 is a creep function, and 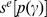 is the elastic response [45], [46]. Finally, it should be noted that all the linear and nonlinear arterial wall models described above can be expressed within the unified framework of the QLV theory, see Table 1.

**Table 1:** Elastic and viscoelastic models of arterial wall strain.

Model	Elastic response 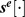	Creep 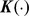	Type
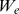	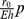	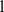	elastic
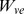	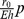	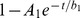	viscoelastic
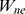	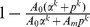	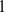	nonlinear elastic

The unified QLV formulation in (8) encompasses all models studied here. The first column lists the model, the second the elastic response, the third the creep, and the fourth states if the model is linear or nonlinear.

#### Mechanoreceptor stimulation

The BR nerves emanating in the adventitial layer of the aortic arch and carotid arteries form a complex branching network [51]. In rats electron microscopy studies have revealed that BR aortic nerve fibers form bundles, usually containing one myelinated and five unmyelinated fibers of different sizes [51, p. 401]. Each bundle is surrounded by a protective sheath, perineurium. Both unmyelinated and myelinated fibers are sheathed in Schwann cells and are embedded in collagen, see [51, p. 404] and [52], [53]. Because these nerve endings are embedded in the arterial wall, deformations of the arterial wall also deform the nerve endings. This stimulates stretch sensitive, non-selective cation channels that serve to transduce the changes in the nerve ending structure into an electrical signal, which is encoded into the firing pattern of the BR neuron [2].

**Figure 4:**
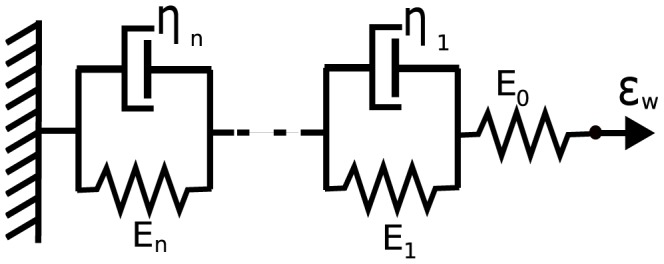
A schematic illustration of the strain sensed by the mechanoreceptors. The spring and 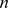 Voigt bodies (a parallel spring and dashpot) in series shown here describes the strain sensed by the mechanoreceptors relative to the deformation of the arterial wall. The spring 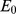 represents the elasticity of the BR nerve endings, whereas the 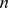 Voigt bodies reflect the viscoelastic properties of the surrounding connective tissue. Each element 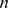 provides a timescale adaptation of BRs firing rate in response to a step increase in pressure observed in experiments. This study compares the cases 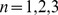.

We propose a model specifying the strain effected specifically at the nerve endings as a result of a given arterial wall strain. Thus our model seeks to capture the stimulation of the mechanoreceptive nerve endings by capturing the stretching dynamics of the nerve endings as the arterial wall expands or contracts in response to changes in pressure. We propose models with the assumption that viscoelastic properties of BR nerve ending connective tissue are the key factor in the transduction process [54], [55]. Following the ideas used in previous BR modeling studies [13], [56]; and before in the modeling of the muscle spindle dynamics [57], [58] we describe the coupling of the strain sensed by the mechanoreceptors to the wall deformation using 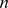 Voigt bodies in series with a spring (Figure 4). Following this idea, the strain sensed by the mechanoreceptors in response to the arterial wall deformation is given by
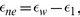
(9)where 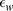 denotes the strain of the wall, and 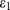 denotes the strain of the first Voigt body. Choosing the parameters 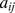 and 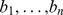, determined by the spring, 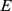, and dashpot, 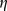, constants, the model given in Figure 4 can be described using the dynamical system
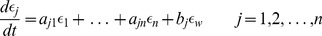
where 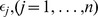 is the relative displacement within each Voigt body. Consequently, our model assumes a declining afferent sensory activity during constant intensity stimulation, a fundamental property of mechanoreceptors that can be described in terms of viscoelastic relaxation processes in the vessel wall [31], [59]. Below we describe, in more detail, the computational aspects of this element of the BR model, analyzing model components including one, two, and three Voigt bodies. Since the strain is calculated using Voigt bodies, we have denoted this model component as 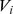 where 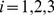 indicates the number of Voigt bodies included.

*One Voigt body model* 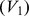. We start with the simplest model, consisting of one Voigt body in series with a spring (Figure 4 for 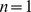). The governing equation used for determining the nerve ending deformation is given by
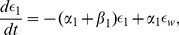
(10)where 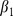 and 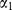 depend on the spring constants 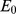, 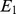 and viscous element 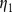 as stated in Table 2. Since equation (10) is a first-order linear ODE, the total strain sensed by the mechanoreceptor is equivalent to the strain on the Voigt body, thus this model component only exhibit one time-scale 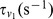 associated with the strain 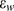. This time-scale is given by
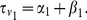
(11)


**Table 2:** The state variables and parameters of the BR models.

Variable	Definition	Units
*p*	aortic blood pressure	mmHg
*<$>\raster(80%)="rg1"<$>_w_*	aortic wall strain	unitless
*<$>\raster(80%)="rg1"<$>*_1_	nerve ending coupling strain 1	unitless
*<$>\raster(80%)="rg1"<$>*_2_	nerve ending coupling strain 2	unitless
*<$>\raster(80%)="rg1"<$>*_3_	nerve ending coupling strain 3	unitless
*<$>\raster(80%)="rg1"<$>_ne_*	nerve ending strain	unitless
*f*	firing rate	Hz

Table 3 contain between three and six state variables listed here. Additionally, the parameters for the whole family of BR models together with their nominal values, units and literature references are provided. The models considered in this work and defined in

*Two Voigt body model* 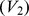. The model with two Voigt bodies and a spring in series (Figure 4 for 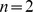) can be described by the following system of equations
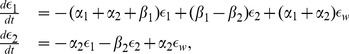
(12)where 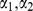, 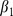 and 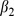 are defined in Table 2. There are two timescales 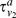 and 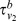 associated with the nerve ending relaxation, thus one expects the BR firing rate to observe adaptation more closely. For this model represented by two Voigt bodies 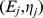, 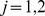, in series with a spring 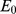, those two time-scales can be computed as follows. The total strain-stress relationship is given by
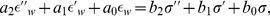
(13)where the coefficients are
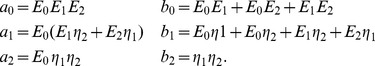
For the step-increase in pressure (and thus wall stain 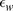) we obtain 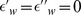. Therefore the two timescales 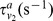 and 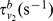 are given by the roots of
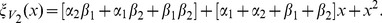
(14)


*Three Voigt body model* 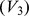. For model with three Voigt bodies in series with a spring (Figure 4 for 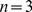) we obtain the following system of equations
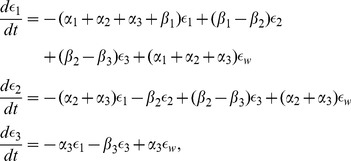
(15)where as in the previous case the coefficients 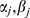, 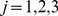 are provided in Table 2. This model has three time-scales 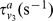, 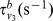, and 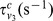 associated with the nerve ending relaxation. Again, the total strain-stress relationship for our model is given by
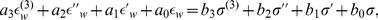
(16)where the coefficients are given by
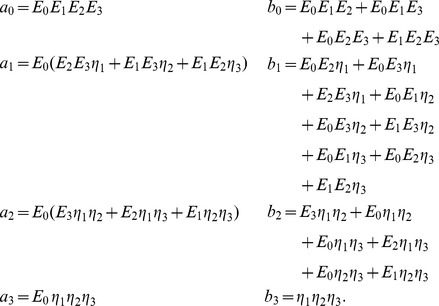
For the step-increase in pressure (and thus wall stain 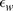) w obtain 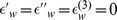. Thus the timescales are the roots of
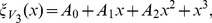
(17)where
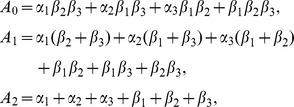
where again 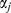, 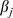 (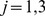) are given in Table 2.

#### BR firing rate

The final model component requires a description of the generation of action potentials in response to stimulation of the mechanoreceptors. The generation of action potentials is often described using the Hodgkin-Huxley (HH) model representing the biophysical characteristic of cell membranes, including a lipid bilayer represented by a capacitance and membrane channel proteins represented as nonlinear resistors. Action potentials are initiated when the neuron receives sufficient electrical current stimulus, in case of BRs, this stimulus is typically via pressure dependent stimulation of stretch sensitive ion channels. These detailed models are fairly complex and contain numerous parameters; moreover, they describe the dynamics of membrane voltage instead of directly modeling firing rate. In this study, we proceed by considering two models: a simple model, that predicts firing rate linearly from the mechanoreceptor stimulation, and using a leaky integrate-and-fire model. The linear model simply amplifying the strain is denoted by 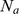, and the integrate-and-fire model is denoted by 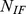.

*Simple amplifier (* 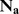 *)*. For the simplest possible model, we assume that action potential generation, and thus nerve firing rate, can be obtained by considering a simple linear amplifier described by
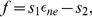
(18)where 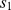 is the *gain*, and 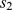 is the *shift*. The underlying assumption of this model is that the change in firing is proportional to the mechanical stimulation, 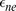, of the nerve ending.

**Figure 5:**
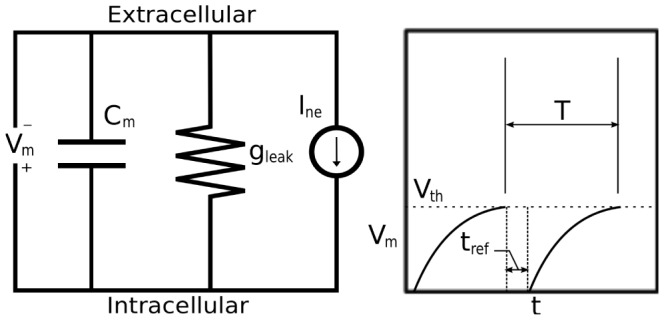
Diagram for leaky integrate-and-fire model. The circuit diagram (left) represents the schematic layout of the integrate-and-fire components. The graph (right) depicts voltage vs time for a neuron stimulated by a constant current.

*Leaky integrate-and-fire model (* 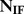 *)*. A more realistic description can be obtained using a leaky integrate-and-fire model, which considers the BR neuron as a simple electrically excitable membrane stimulated by a current generated by the mechanoreceptors. We assume that the generated current is proportional to the strain sensed by the nerve endings 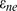. The leaky integrate-and-fire model originally proposed by Lapicque [60], but also discussed in [61], [62], describes the excitation of the voltage across the BR membrane as equivalent to the capacitor voltage in an RC circuit (Figure 5). The circuit consists of a stimulus current source (given as a function of 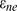), an Ohmic leakage conductance, 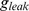, and a capacitor, 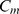, all three elements in parallel.

The change in voltage generated by a leaky integrate-and-fire model is given by
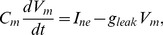
(19)where 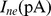 denotes the current stimulus, 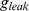 (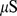) is a leakage conductance, and 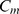 (nF) denotes the membrane capacitance. In the equation above, the voltage 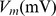 is relative to the equilibrium potential. To model the firing rate of the neuron we assume that to form an action potential, the BR neuron has to charge the membrane voltage above a given voltage threshold, which we denote 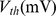. Applying this assumption to (19), allows calculation of 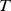 (s), i.e., time required for the voltage to increase from equilibrium to the threshold, for a given stimulus current, 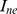. We can calculate 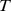 integrating (19) giving
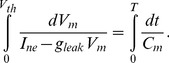
(20)For constant 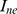 this equation can be solved analytically, yielding
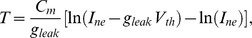
where, as stated above, 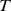 represents the time required to generate an action potential given a constant current stimulus 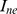. We propose to model 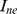 as a linear function of 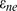


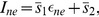
(21)where 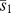 and 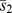, both of units (s), are the *gain* and *shift* of the stimulus current. Finally, the absolute refractory period, 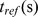, denotes the time following an action potential, during which a subsequent action potential cannot be generated [63]. We account for this by letting the rate (frequency) 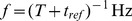. With these simplifying assumptions the BR firing rate can be computed as a function of the instantaneous strain of the nerve ending sensed by the BR as
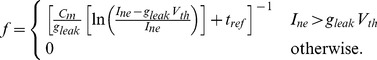
(22)We propose to interpret the BR firing rate as that given by (22) for 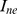 at a given instant. The piecewise definition of the frequency is necessary as (20) does not have a solution when the stimulus current is less than the leak current at threshold voltage. This is a consistent interpretation of the instantaneous frequency as we do not expect any firing events to occur for a sub-threshold stimulus (less than the base current). In general, for a sub-threshold current stimulus the firing rate, 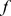, is expected to cease until 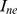 is increased above the threshold level. The parameters 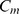, 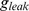, 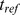, and 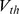 of this model are expected to approximately correspond to the electrophysiologically observable characteristics of the BR neuron, membrane capacitance, leakage conductance, refractory period and threshold, respectively. The membrane capacitance can be measured using electrophysiological techniques [64]. Leakage conductance can be approximated as the net inward conductance near equilibrium potential. The true refractory period and threshold voltage of a neuron are not absolute and are typically somewhat dynamic and thus difficult to measure. One may roughly estimate these values for BRs from the results of experimental studies of the membrane excitability of nodose neurons, a neuron family including BRs [64]. The observation of BR firing rates up to 140 Hz leads to a refectory period of 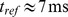 [21], [22].

#### Composite BR models

In the previous sections we developed a framework to model the three main components involved with description of the BR firing. To develop a composite model, one component must be chosen from each category. There are various options one may select from in order to construct a BR model. The choice depends on a number of factors including the type of species (e.g., rats, dogs, sheep, humans, etc.) and the type of data (e.g., steady, step-change, dynamic, *in vivo*, etc.). We propose a total of six linear and nonlinear models, summarized in Table 3, which we will carefully analyze and test using aortic baroreceptor rat data. These models can be formulated as a system of algebraic and differential equations of the form
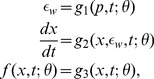
(23)where 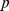 (mmHg) is the blood pressure (model input); 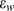 denotes the vessel strain; 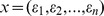; 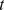 time (s); 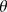 the model parameters; and 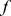 (Hz) the BR firing rate. Models can be classified as one of two basic types: linear and nonlinear models. It should be noted that differential equations only enter via the model component describing mechanoreceptor strain. To ensure that model simulation began from a relaxed state, we computed the initial conditions by solving 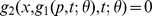. To be more precise for the four linear BR models 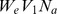, 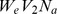, 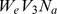, and 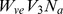 the initial conditions are respectively
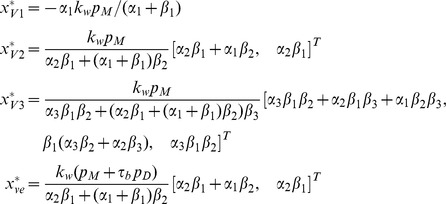
where 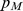 and 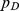 (mmHg) are the initial values of the pressure stimulus and its derivative, respectively, and 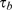 (s), 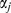 (mmHg), 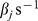, for 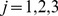 are given in Table 2. For the nonlinear model 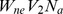 we used the following initial condition
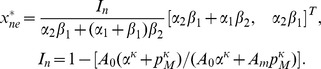



**Table 3:** Summary of the BR models.

Model	Wall	Nerve ending	Neuron	Parameters
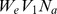	Eq (4)	Eq (10) & (9)	Eq (18)	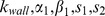
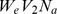	Eq (4)	Eq (12) & (9)	Eq (18)	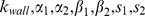
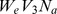	Eq (4)	Eq (15) & (9)	Eq (18)	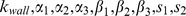
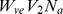	Eq (7)	Eq (15) & (9)	Eq (18)	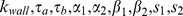
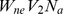	Eq (5)	Eq (15) & (9)	Eq (18)	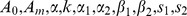
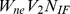	Eq (5)	Eq (15) & (9)	Eq (22)	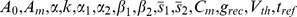

[29]. Each model is denoted by a three-element name referring to a corresponding part of its component (arterial wall 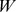, mechanoreceptor stimulation 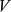, neuron 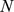). The cross-reference indicates what equation is included in a given model. The table defines six BR models that are tested against previously recorded BR data from rats

## Results

In this section we present results obtained with the models introduced in the Method section and summarized in Table 3. First, we test the models' abilities to quantitatively fit experimental data with sinusoidal and step-increase stimuli. Second, we discuss the models ability to show qualitative features not encompassed by the quantitative data. Quantitative simulations allow us to identify the components necessary to fit observed data, whereas qualitative simulations allows us to test the model further in response to stimuli not detailed by experimental measurements.

### Quantitative results

Models will be tested quantitatively using three types of pressure stimuli: sinusoidal at a fixed frequency, a step-increase, and a step-increase followed by a step decrease (Figure 2A–C). We investigated six linear and nonlinear models summarized in Table 3. For the wall strain three models were investigated, the simplest assumes the wall strain 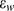 has a spring-like response (denoted 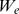). The second model (denoted 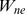) accounts sigmoidally for increased stiffening with increased pressure, and finally we investigate a viscoelastic model (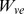). The mechanoreceptor strain 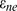, is modeled using one, two, and three Voigt bodies, respectively, in series with the spring (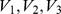). Finally, two models were used for determining the BR firing rate, a linear model (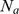) and an integrate-and-fire model (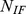). As mentioned above, these models can all be described as a system of algebraic and differential equations. For all models the model input is pressure 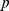 and the model output is BR firing rate 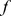, initial conditions were computed to ensure that model solutions start at steady state. The objective was to estimate model parameters minimizing the least squares error between the model and data. This is calculated from the point wise residual error between model and data
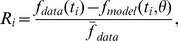
where 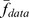 is the average firing rate of the specific data set considered and 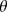 denotes the parameter vector. To estimate the parameters we minimize the sum of squares cost function (referred to as RMSE in Tables 4, 5, and 6)
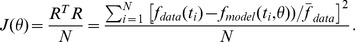



**Table 4:** Optimized values of parameters for the linear models of BR response.

	Data	*k_wall_*	*α*_1_	*α*_2_	*α*_3_	*β*_1_	*β*_2_	*β*_3_	*S*_1_	*S*_2_	*p*_1_	*p*_2_	*θ*	*R* ^2^	RMSE
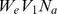 IC	sine	0.0063	0.5			0.5			480	100	6.45	46.75			
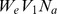 opt	sine	0.0063	0.5			0.5			1076	375	6.44	46.84		**0.949**	**2.522**
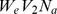 IC	sine	0.0063	0.5	0.4		0.5	2		480	100	6.45	46.75			
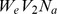 opt	sine	0.0063	0.5	0.4		0.5	2		1105	346	6.44	46.89		**0.950**	**2.507**
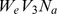 IC	sine	0.0063	0.5	0.4	1	0.5	2	10	480	100	6.45	46.75			
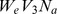 opt	sine	0.0063	0.5	0.4	1	0.5	2	10	1221	333	6.44	46.95		**0.951**	**2.495**
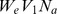 IC		0.0063	0.5			0.5			480	100					
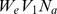 opt	step 1	0.0063	0.522			0.395			360	104			1.090	**0.899**	**1.860**
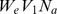 opt	step 2	0.0063	0.273			0.407			340	140			1.025	**0.919**	**2.677**
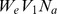 opt	step 3	0.0063	0.241			0.438			378	169			1.025	**0.969**	**2.420**
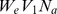 opt	step 4	0.0063	0.273			0.865			398	201			1.055	**0.983**	**1.832**
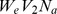 IC		0.0063	0.5	0.4		0.5	2		480	100			1		
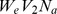 opt	step 1	0.0063	0.398	0.4		0.310	2		376	102			1.097	**0.905**	**1.800**
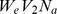 opt	step 2	0.0063	0.188	0.4		0.304	2		365	137			1.027	**0.917**	**2.702**
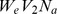 opt	step 3	0.0063	0.132	0.4		0.271	2		480	163			1.099	**0.970**	**2.390**
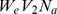 opt	step 4	0.0063	0.101	0.4		0.552	2		480	201			1.057	**0.983**	**1.823**
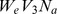 IC		0.0063	0.5	0.4	1	0.5	2	10	480	100			1		
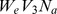 opt	step 1	0.0063	0.415	0.4	1	0.303	2	10	404	102			1.100	**0.908**	**1.779**
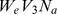 opt	step 2	0.0063	0.208	0.4	1	0.305	2	10	397	138			1.030	**0.917**	**2.719**
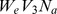 opt	step 3	0.0063	0.135	0.4	1	0.257	2	10	429	163			1.103	**0.970**	**2.426**
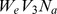 opt	step 4	0.0063	0.101	0.4	1	0.523	2	10	429	201			1.060	**0.984**	**1.810**

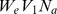
, 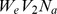 and 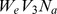 of BR response we present the initial and optimized values of their parameters. We used the BR firing data published by Brown [20] for two different stimuli: the sinusoidal-like pressure profile, and step pressure increase with different magnitude. For the three linear models

**Table 5:** Optimized nonlinear models of BR response: wall strain models.

	*k_wall_*	*A*_0_	*A_m_*	*α*	*k*	*α*_1_	*α*_2_	*β*_1_	*β*_2_	*s*_1_	*s*_2_	*τ_a_*	*τ_b_*	*p*_1_	*p*_2_	*R* ^2^	RMSE
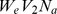 IC	0.0063					0.5	0.4	0.5	2	480	100			6.46	46.75		
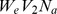 opt	0.0063					0.5	0.4	0.5	2	1105	346			6.44	46.89	**0.950**	**2.507**
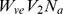 IC	0.0063					0.5	0.4	0.5	2	480	100	0.030	0.01	6.46	46.75		
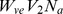 opt	0.0063					0.5	0.4	0.5	2	1112	349	0.028	0.01	6.44	46.77	**0.950**	**2.517**
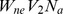 IC		1	3	150	10	0.5		0.5		480	100			6.46	46.75		
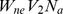 opt		1	32.6	150	10	0.5		0.5		619	109			6.44	46.89	**0.952**	**2.458**

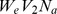
, 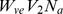, and 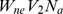, of BR response, we present the initial and optimized values of their parameters. We used the BR firing data published by Brown [20] for a sinusoidal-like pressure profile. For the three models,

**Table 6:** Optimized linear and nonlinear models of BR response: Post-excitatory depression.

	*k_wall_*	*α*_1_	*α*_2_	*β*_1_	*β*_2_	*s*_1_	*s*_2_	*δ_u_*	*δ_d_*	*_R_* ^2^	RMSE
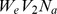 IC	0.0063	0.5	0.4	0.5	2	480	100	4.60	8.70		
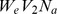 opt	0.0063	0.5	0.4	0.5	2	1076	375	4.59	8.59	**0.862**	**7.384**
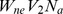 IC	0.0063	0.5	0.4	0.5	2	480	100	4.60	8.70		
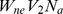 opt	0.0063	0.5	0.4	0.5	2	1076	375	4.59	8.59	**0.883**	**6.795**

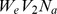
, 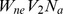 and 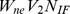 of BR response we present the initial and optimized values of their parameters. We used the BR firing data published by Brown [20] for a square pressure profile. For 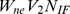 values for 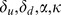 were 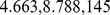 and 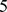 respectively. These are not listed as they were not part of the optimization process for this model. For the three models

Since data is only available for the BR firing rate and the pressure stimuli, for most models not all parameters are identifiable. We denote as identifiable parameters, those that are sensitive and not correlated, given the model output and the associated available data [65]. In this study, identifiability of parameters was determined using sensitivity based methods [66]. Subsequently, for models completely characterized by smooth functions, the Levenberg-Marquardt method was used to estimate model parameters, while for models not fulfilling this requirement (the integrate-and-fire models), parameters were estimated using the Nelder-Mead method. Both used optimization algorithms from Kelley [67].

Below we first describe the methodology used for sensitivity analysis and parameter identification and subsequently we discuss results obtained using nonlinear optimization, the latter is separated according to the input stimulus.

**Sensitivity analysis:** For any smooth model of the form (23), the sensitivities [68]–[70] can be computed as
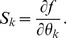
Following Pope et al. [71], we use a finite difference approximation to compute 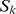


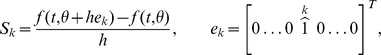
where 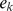 is the unit vector in the 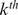 component direction and 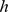 is a small number. The BR firing rate 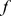 is obtained computationally, with an integration tolerance of 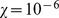 imposed on solution of the differential equations, thus 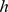 is bounded by 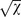. To satisfy this requirement we let 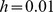.

Sensitivities are ranked by averaging time-varying functions using the two-norm. For each model, this ranking was used to separate parameters into two groups: one group consisted of parameters to which the model output was sensitive, and the other group consisted of parameters to which the model output was insensitive. Estimating only sensitive parameters allows more reliable estimation of parameters [72].

Not all sensitive parameters are practically identifiable [65], [66]. To identify parameter correlations, we used the QR-SVD subset selection method [71], [73], [74]. We also used a method based on covariance analysis to identify pairs of correlated parameters [66]. For each pair of correlated parameters the least sensitive parameter was kept fixed at its nominal value while the other was included in the subset. Parameter correlations were computed from
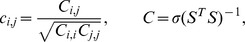
where 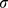 is the variance of the assumed noise in the data, 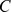 is the covariance matrix, and is 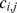 the correlation coefficient. Parameters for which 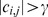 are labeled as correlated. For the models studied in this work we let 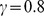. Once a set of uncorrelated sensitive parameters were identified, we used either the Levenberg-Marquardt or the Nelder-Mead method to estimate the subset of practically identifiable model parameters [67]. The Levenberg-Marquardt method was used for models that can be described using smooth functions, while the Nelder-Mead method was used for models including the leaky integrate-and-fire component. Since this model contains a discontinuity the gradient based Levenberg-Marquardt method is not applicable.

**Figure 6:**
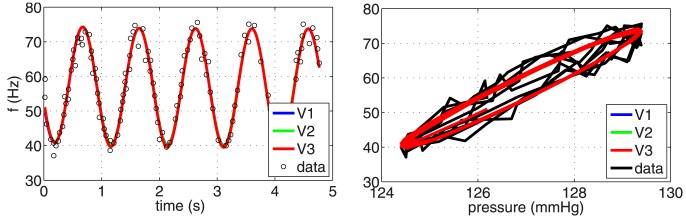
The optimized response of linear BR models (left), and the corresponding hysteresis loop (right). We present the fits for three linear BR models 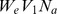, 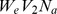 and 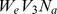 (denoted in the legend as V1, V2, and V3, respectively), listed in Table 3. The optimized parameter values, the 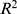 and the RMSE errors are reported in Table 4.

*Sinusoidal stimulus*: Now we present results obtained using sinusoidal forcing allowing us investigate asymmetry of the model response. Results (Figure 6) show BR firing rate as a function of time and BR firing rate as a function of stimulus. For both graphs model results are marked with red lines and data with black. The associated pressure stimulus is depicted in Figure 2A. For this stimulus we analyzed five models. We first describe results obtained with the three linear models, analyzing the impact of including one, two, or three Voigt bodies. Second we discuss results obtained with the nonlinear models analyzing the impact of including more advanced description of the wall strain. For this stimulus we did not analyze the integrate-and-fire model, since we did not anticipate any added effect of this model because of the input rage of the pressure stimulus.

The three linear models include a component determining the wall strain, described using a linear elastic function of pressure, a component representing mechanoreceptor stimulation, described using one, two, and three Voigt bodies, and a component predicting the BR firing rate. The three models have 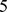, 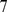, and 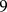 parameters, respectively, as well as two additional parameters 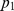 and 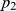 associated with the sinusoidal stimulus. In [20, p. 695] the authors indicated that phase measurements are less accurate than amplitude measurements due to the inaccuracies associated with assigning interspike intervals to bins. Thus, the parameters 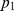 and 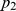 were added to the parameter set. Sensitivity analysis together with subset selection allowed us to identify four uncorrelated parameters including 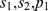, and 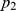, which were estimated for all three models.

The nominal values for the model parameters (listed in Table 4) were computed as follows. The parameter 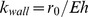 (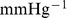), where 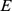 is Young's modulus (mmHg), 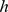 (mm) is the wall thickness, and 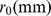 is the zero pressure radius as described in the Methods section (see also Table 2). We use 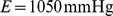 approximating a lower bound to values observed in a previous study [75]. In [76, Figure 1] Feng et al. provide detailed measurements of the external diameter 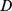 and thickness 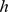 for the rat aortic arch, measured in adult male Sprague-Dawley rats. They found that in the region with aortic BR endings the average values of 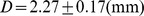 and 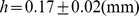. Using these values we compute 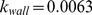 (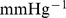). We note this parameter and 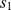 were highly correlated indicating equivalent fits could be achieved through adjustment of either parameter. No direct experiments exist allowing estimation of nominal values for the elastic 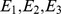 and viscous constants 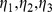 associated with mechanoreceptor strain. These parameters appear only in the dynamic part of the model and determine the adaptation time-scales. To ensure that the three models are distinct, it is essential that parameters representing time-scales are separated, otherwise the models would essentially reduce to one. This knowledge, along with values chosen in the study by Bugenhagen et al. [56] motivated our choice for nominal parameter values. To avoid the problem of structural nonidentifiability [65] we rescaled the parameters as follows 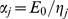 and 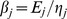 for 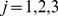. The full list of the model parameters together with their initial conditions, units and literature reference is provided in Table 2. As for the stimulus, the average pressure (127 mmHg) and the amplitude (5 mmHg) was provided in [20]. To compute the frequency 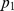 and the shift 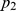 of the pressure, we digitized the stimulus provided in [20], Figure 2A, and then fitted to a sinusoidal function 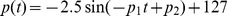, obtaining 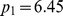 and 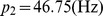. As noted in Figure 6, results of parameter estimation with each of the three models were indistinguishable, though estimated parameter values varied significantly, the latter is due to added complexity associated with adding more Voigt bodies. The fact that graphs were almost identical was also reflected by the least squares cost RMSE (and the coefficient of determination 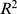) for models 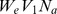, 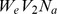 and 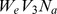 we obtained 2.522 (0.949), 2.507 (0.950), and 2.495 (0.951), respectively, see Table 4.

Next, we investigated the impact of including more complex wall models. Additionally, we incorporated a nonlinear response wall model 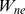, and a viscous wall model 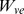. To be more precise we compare the BR response of the following three models 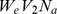, 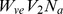 and 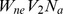 described using 7, 8, 9 parameters plus the two parameters associated with the stimulus. We examined the ability of each of these models to fit the sinusoidal stimulus. Sensitivity analysis and subset selection allowed us to estimate 4–6 parameters. All models allowed us to estimate 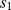, 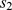, 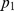, and 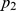. In addition, for the nonlinear elastic model 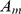 was added to the subset and for the viscoelastic model 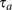 and 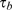 were added to the subset. Given that the more complex nonlinear models allows estimation of more parameters, one should anticipate better results. But due to the limited dynamics embedded within the pressure stimulus, adding more complex wall models did not improve results as reflected by the least squares cost RMSE (and the coefficient of determination 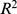), which for 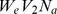, 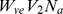 and 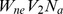 gave 2.507 (0.950), 2.517 (0.950), and 2.458 (0.952), respectively; see Table 5.

*Step-increase stimulus*: This section presents results with the same five models previously used for prediction of the BR response with the sinusoidal pressure stimulus. As with the sinusoidal stimulus we do not test the integrate-and-fire model, due to the nature of the input stimulus. Again, we first discuss results obtained with the three linear models 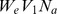, 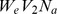 and 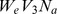 followed by results obtained using the more complex nonlinear and viscoelastic wall models.

**Figure 7:**
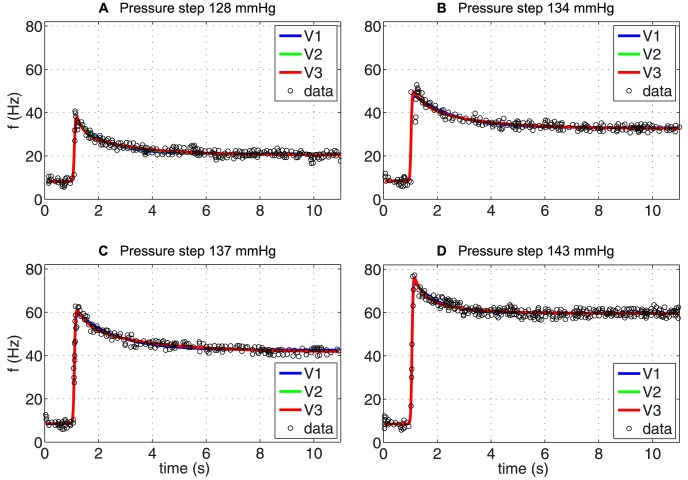
The optimized response of linear BR models. We show the ability of three linear models 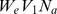, 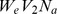 and 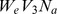 (denoted in the legend as V1, V2, and V3, respectively) to reproduce four types of increases in pressure: ((A) 128 mmHg, (B) 134 mmHg, (C) 137 mmHg, and (D) 143 mmHg) published by Brown [20]. The parameters of each model have been optimized for each data set individually and are listed in Table 4 together with the 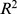 and the RMSE errors.

Studies were done to capture the effect of overshoot and adaptation in response to four input stimuli varying in the magnitude of the pressure step. All stimuli start at the same baseline pressure, and the step-increase was imposed at the same time 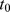. As before the three models have 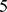, 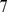, and 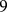 parameters, respectively, but functions describing the “smooth” step pressure increase (2) only involve one additional parameter 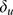, representing the onset of the step-increase. This parameter was not provided in [20]. Subset selection together with efforts to make model comparison possible resulted in 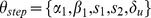. As reported in [20, Figure 5] the baseline pressure associated with the step-increase stimulus was set to 115 mmHg, and the step-increases (from the baseline) to 128, 134, 137, 143 mmHg, respectively. Figure 7(A–D) shows the ability of the three linear BR models to reflect observed overshoot and adaptation. Each panel shows the optimized firing rate. The least squares cost RMSE (and the coefficient of determination 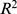) of model 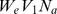 for the optimized values of its parameters with respect to the four step-increases 128, 134, 137, and 143 mmHg were: 1.860 (0.899), 2.677 (0.919), 2.420 (0.969), and 1.832 (0.983). Marginal improvements were obtained with 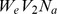, which gave: 1.800 (0.905), 2.702 (0.917), 2.390 (0.970), and 1.823 (0.983), and finally, for 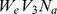 the values were: 1.764 (0.909), 2.700 (0.918), 2.390 (0.970), and 1.809 (0.983), see Table 4. Similar to the sigmoidal stimulus, no improvements (results not shown) were obtained with the more advanced nonlinear and viscoelsatic wall models.

**Figure 8:**
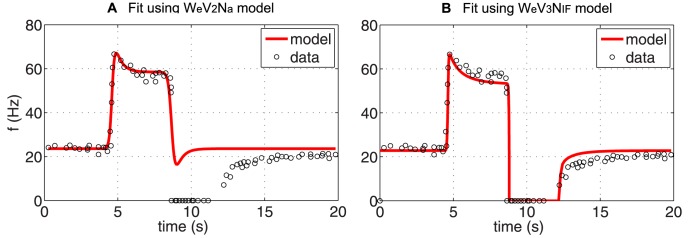
The optimized response of (A) 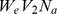, and (B) 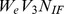 to a PED profile of BR firing rate. The parameters of each model have been optimized for each data set individually and are given in Table 4 together with the 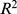 and the RMSE errors.

*Square stimulus*: The square stimulus is characterized by a constant pressure input followed by a step-increase after which the pressure is decreased to its baseline value. This type of stimulus primarily tested the models' ability to reflect PED followed by recovery, although other features including adaptation and overshoot are also shown. Similar to previous studies we first investigated the simpler linear models including one, two and three Voigt bodies. For the square input stimulus, in Figure 8A, we plot BR firing rate data extracted from Saum et al. [32] (circles) and the corresponding optimized fit using 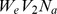 (solid line), changing the number of Voigt bodies did not improve the model response. This model has 7 parameters and additional two 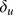 and 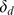 related with the input stimulus (3). Subset selection together with our effort to make model comparisons possible made us estimate the parameters 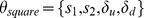. The least squares cost RMSE (and the coefficient 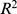) with optimized parameters was 7.384 (0.862 for 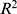), see Table 6. While the model, as anticipated, was able to produce overshoot and adaptation, this model was not able to capture PED accurately.

We hypothesize that the inability to show PED is due to the simple linear firing rate model, which does not allow the BR firing rate to cease for sub-threshold stimuli. Thus, we first investigated the impact of exchanging the linear BR firing rate model with the integrate-and-fire model. Including the integrate-and-fire model clearly improved results (not shown) though with the linear wall model it was difficult to accurately fit the data both during adaptation and recovery. Subsequently, we analyzed the impact of exchanging the linear wall model with the nonlinear wall model, keeping the integrate-and-fire model. Results with this model (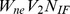) is shown in Figure 8B. This figure shows the recorded BR firing rate (circles) and the model fit (solid line) in response to the square pulse stimulus. Model parameters estimated include 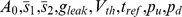. Optimized parameter values and units are given in Table 6 together with the 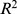 and RMSE errors. Finally, we investigated the impact of adding a viscoelastic wall model, which did not provide any additional improvements.

**Figure 9:**
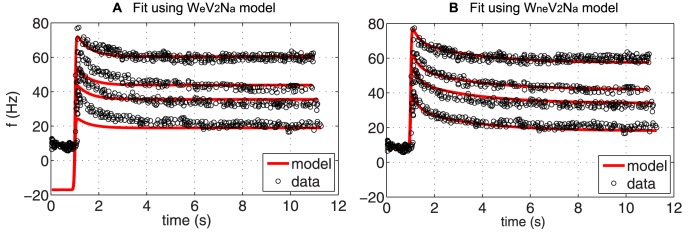
Simultaneous response with a linear and a nonlinear BR model. (A) Predictions obtained estimating one parameter set for all four pressure step-increases using the linear model with two Voigt bodies 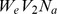. Note, that the overshoot is diminished for responses to smaller step-increases in pressure, and that the baseline firing rate is not reproduced accurately. (B) Predictions obtained with the nonlinear model 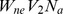 accounting for nonlinear stiffening with increased pressure allowed us to accurately fit all four responses using one set of parameter values.

*Simultaneous fits*: Figure 7 showed that linear models can exhibit overshoot, adaptation, and can fit the firing rate data for all four step-increases, though as reported in Table 4, each step-increase resulted in significantly different parameter estimates. However, data are extracted from experiments done within the same fiber, thus we expected only small variation in parameter values. We performed additional optimizations to investigate if the observed differences in the parameter estimates, were simply a result of performing optimizations for one stimulus at the time. To remedy this problem, we estimated one set of parameters for all four step-increases. Results of this simulation are shown in Figure 9A (computed with the model 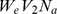). This simulation confirms that the simple linear model cannot estimate one set of parameters that allows *simultaneous* fit of the response to all four pressure stimuli. Similar results were obtained with the other models. In particular, it should be noted that the overshoot is diminished for the smaller step-increases, and that the model was unable to capture the correct baseline firing rate. In contrast, when including a nonlinear elastic wall 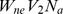 we were able to estimate one set of parameters that allowed us to simultaneous fit the response to all four pressure stimuli. This model accurately reproduced the baseline firing rate as well as the overshoot and adaptation observed in response to the step-increase (Figure 9B). We hypothesize that this difference is due to larger range of pressure within the applied stimuli, where the known nonlinear behavior of the arterial wall deformation plays an important role. It is known that arteries appear stiffer at higher pressures than at lower pressure. Thus the nonlinear wall model significantly improves the fit.

### Qualitative results

In the previous section we showed the ability of our proposed linear and nonlinear BR models to fit the firing rate data measured from rats. It is well known (see section Methods) that the BR firing rate can exhibit a number of qualitative characteristics including saturation, threshold, adaptation, overshoot, PED and rectification. The quantitative data used to test the model in the previous section showed adaptation, overshoot, and PED, in response to a sinusoidal (with fixed amplitude) and step changes (increase/decrease) in blood pressure. However, these stimuli did not test saturation, threshold, or rectification. Although the models show adaptation, no clear conclusion could be drawn to determine how many Voigt elements (time-scales) were needed to reflect known BR firing rate observations.

Now we show our preferred model 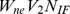 with estimated parameters, including nonlinear deformation of the elastic wall, two Voigt bodies for computing nerve ending stimulation, and a leaky integrate-and-fire model for predicting firing rate, exhibits the features not yet studied experimentally. This was done using ramp and sinusoidal (with varied amplitude and frequency) pressure stimuli.

**Figure 10:**
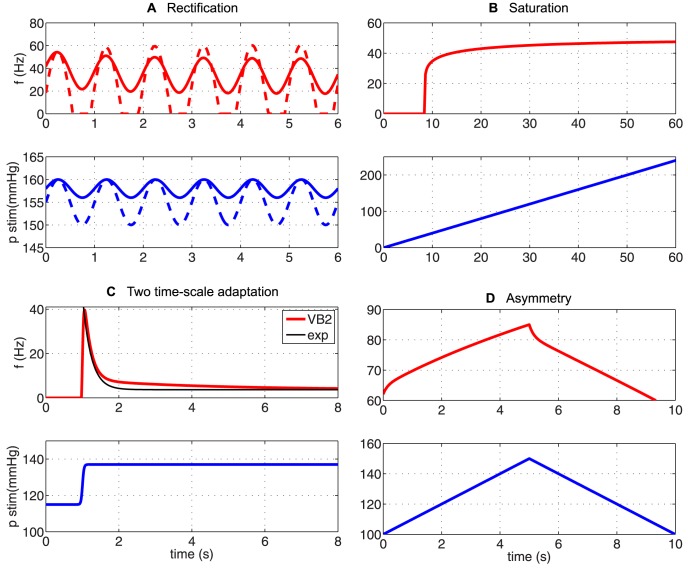
Qualitative responses. We present a qualitative response of the two Voigt body BR model 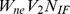 to various pressure stimuli including sinusoidal (A), ramp up (B), step-increase (C), and trianglular (D) showing the model's ability to reflect rectification (A), saturation (B), two time-scale adaptation (C), and asymmetry (D).

*Rectification*: Figure 10A presents the model's response to a sinusoidal wave pressure stimulus with various amplitude. This simulation is motivated by the observation of Brown et al. [20, Figure 6] that a 2.5 increase in amplitude of the sinusoidal stimulus resulted in an increased amplitude of the firing rate, with a lower mean firing rate. Moreover, it was noted that for large amplitude stimulation the firing rate ceases during the trough of the pressure wave. These two observations are referred to as rectification. One could question if the simpler linear model is able to display this phenomena. The linear wall model would certainly be able to reproduce the increased amplitude for a single stimulus, but again, if multiple stimuli were tested, correct predictions require the nonlinear wall model. Moreover, the ability of the firing rate to cease requires the threshold built into the integrate-and-fire model. With the simple linear neuron model, the firing rate would become negative, which does not represent what happens physiologically.

*Threshold and saturation*: Two other prominent firing characteristics are threshold and saturation. In [27, Figure 5] Seagard et al. noted that BRs with a higher threshold pressure were less sensitive, had lower discharge rates, and had higher values for saturation. Receptors with higher discharge rates were also more sensitive and were found to have afferent fibers with greater conduction velocities. In Figure 10 B we show that our model 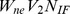 is able to reproduce qualitatively similar saturation features.

*Adaptation*: Even though our quantitative models were able to capture adaptation, it was noted that results with one, two, or three Voigt bodies were similar, in other words, the models could not clearly distinguish if the adaptation process included one or three time-scales. Yet, several authors (e.g., [11], [12], [30], [77]) have hypothesized that adaptation occurs with more then one time constant. It is also known that the muscle spindle can produce a response of this kind to a clipped-off ramp stretch [78]. Figure 10 C shows that the studied model 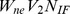 admits the fast adaptation and the slow adaptation in agreement with experiments. We also plot an exponential fit and show that a similar adaptation is not possible by only one exponential function. This qualitative feature made us include two Voigt bodies in our preferred model, a conclusion that could not have been made strictly from quantitative simulations presented in the previous section.

*Asymmetry*: In Figure 10 D we show that our preferred model 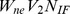 clearly exhibits asymmetry when exposed to a ramp-up followed by a ramp-down pressure stimulus, which agrees with experiments (see e.g., [23]).

## Discussion

The objective of this study was to develop a *mathematical framework* for constructing computationally efficient and accurate BR models, which in contrast to the existent models, are able to reflect *all* known qualitative BR firing features as well as fit quantitative data. Our overall aim was not to focus on a concrete experimental species but rather to formulate a family of BR models, which could potentially be included in a more comprehensive model of CV system. Quantitative computations were done comparing our models to experimental measurements by Brown et al. [20] and Saum et al. [32]; while qualitative studies were performed to show that our preferred generic model 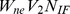 is able to exhibit all known firing rate responses. All models used blood pressure as an input and computed the BR firing rate as an output. Although our procedure was designed to be generically applicable to various species and multiple types of baroreceptors, we tested our models using only quantitative data from experiments preformed using aortic baroreceptors from rats.

We believe that this is the first work that offers a systematic approach to building and evaluating BR models with the objective to provide the simplest possible family of generic models. Our modeling framework first analyzed the known physiology and common features of the firing rate observed in the BRs of various species. Second we generated submodels describing each stage of the physiological response: arterial wall deformation, stimulation of mechanosensitive channels found in the BR nerve endings, and generation of action potentials. Finally we modeled the BR system by combining the submodels in various configurations (summarized in Table 3). Each of these configurations was tested in order to determine the contributions of each component to the transduction of the BR signal. This process allowed identification of the importance of nonlinear effects of two critical sub-systems in the BR response, the arterial wall and the neuron itself. This framework advanced the state of BR modeling by first evaluating models comparatively with respect to the same data and features, second by generating a model which fits all known characteristics of BR firing qualitatively, and third by developing a model which is capable of fitting multiple data sets of BR firing rates quantitatively.

A particular insight was revealed by consideration of BR models with various descriptions of the arterial wall. Applying our framework demonstrated the insufficiency of linear wall models' representations of the response of a single BR neuron to multiple step-pressure inputs (see Figure 9A). A nonlinear elastic wall model was required to implement a model capable of accurately fitting the BR response to multiple pressure levels with one set of parameter values (see Figure 9B). The choice of this model is further motivated by the well known fact that arteries exhibit nonlinear deformation with saturation at both high and low pressures [23], [27]. Additionally by applying our framework and considering the effects of including the viscoelastic wall model, we found that the additional complexity did not contribute to better definition of BR dynamics, despite previous studies having shown wall deformation does have viscous components [45], [79]. This is likely due to our modeling choice for nerve ending stimulation. This portion was modeled using two Voigt bodies in series to allow adaptation at multiple time-scales. Data is not available to separate the viscoelastic part of the wall-deformation with the viscoelastic deformation associated with stimulation of the mechanosensitive channels, thus indirectly our model exhibits both features. One explanation would consider the first Voigt body to be associated with wall deformation while the second is associated with nerve ending deformation. Moreover, it should be emphasized qualitative simulations were needed to show that the two Voigt bodies allow multiple time-scales, a feature we were not able to extract from simulations alone. These considerations, and our studies, affirm the importance of viscoelastic effects; however, in terms of simplicity it is advantageous to isolate the viscoelastic components within the model, and further we note linear viscoelastic effects are sufficient to capture the dynamics of BR firing when coupled with a nonlinear elastic total deformation of the arterial wall.

To our knowledge, this study provides the first direct measure of the importance of incorporating various time-scales in BR models. It is believed that various time-scales in the adaptation process are due to the viscoelastic coupling of the nerve ending to the arterial wall. We chose to emphasize this in our modeling process by considering different numbers of Voigt bodies in series with a spring. In Table 4 we show the results of testing three models 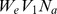, 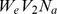, and 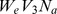 differing only with respect to their nerve ending models 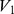, 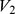, and 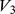, respectively. Our findings indicate that no more then two timescales in the adaptation process are needed in order to achieve a very precise fit to the data. This conclusion is closely related to the fact that we tested our models using rat data with fairly limited pressure-stimulus response as only this type of experiments are currently available. To test this component more carefully, it is essential to analyze data recorded over longer time-scales.

Another insight afforded by this investigation highlights the importance of nonlinearities in the neural response to mechanoreceptor strain. As hypothesized previously [30], our study affirms the nonlinearities of action potential generation, even for the leaky integrate-and-fire model 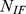 are sufficient to produce the hysteretic phenomenon of PED. In contrast the simple linear model 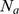 of firing in response to mechanoreceptor strain does not allow for the asymmetric responses seen in PED as well as in the response to sinusoidal stimulus with high amplitude. The nonlinear-elastic wall in combination with two Voigt bodies modeling mechanoreceptor stimulation responds in an equal but opposite manner to rising and falling pressure, thus the change in firing rate with the linear model is symmetric to step-increase and step-decrease, which is not reflective of the data. We affirm the hypothesis that the neuron itself is responsible for generating PED, as this feature was robustly represented by the leaky integrate-and-fire model regardless of the mathematical description for arterial wall strain. This would provide a good explanation for the observation of PED in multiple species, many of which have a high degree of variability in the viscoelasticity in their respective arterial walls.

The results and insights generated through application of our proposed modeling framework are not limited to those presented in this study. In addition it provides a means to identify which features and what level of detail of the underlying physiological systems are of greatest significance in generating BR dynamics. This ability is useful in developing experiments which may be able to isolate physiology responsible for a given phenomenon, such as the responsibility of the neuron in generating PED. Further this approach provides evaluative power to make design decisions when developing a model for a specific data interpretation or simulation task. An example of this follows from our insights into the role of the arterial wall in BR signal transduction. Although the arterial wall may best be modeled using viscoelastic theory, our framework allows a modeling decision to be made in favor of simplicity if only the output dynamics are of interested.

This investigation further suggests a methodology for integrating a model generated in this manner into a model of larger scope. Suppose a mathematical representation of an overall baroreflex system (see Figure 1) is desired to reflect only normal physiological conditions, then it may be sufficient to use only simplified description of the BR signal. For example a simple linear firing rate model may be adequate for simulations operating in the range above the firing rate threshold. However, to reflect heart rate at various abnormal physiological conditions a more complex model combining nonlinear deformation with the leaky integrate-and-fire model may be necessary. Additionally, application of our modeling approach to a larger CV model might reveal features of the BR subsystem with importance in maintaining homeostasis. We hypothesize that overshoot, adaptation and recovery, features of the BR firing in response the extremes of pressure waves, are critical for regulation of blood pressure during stressful situations, such as a head-up-tilt experiment.
